# Quality of fixed dose artemether/lumefantrine products in Jimma Zone, Ethiopia

**DOI:** 10.1186/s12936-019-2872-1

**Published:** 2019-07-15

**Authors:** Sileshi Belew, Sultan Suleman, Tesfaye Mohammed, Yimer Mekonnen, Markos Duguma, Henok Teshome, Bikila Bayisa, Evelien Wynendaele, Matthias D’Hondt, Luc Duchateau, Bart De Spiegeleer

**Affiliations:** 1 Drug Quality and Registration (DruQuaR) Group, Faculty of Pharmaceutical Sciences, Ghent University, Ottergemsesteenweg 460, 9000 Ghent, Belgium; 2 School of Pharmacy, Jimma University, PO Box 378, Jimma, Ethiopia; 3 Biometrics Research Group, Faculty of Veterinary Medicine, Ghent University, Salisburylaan 133, 9820 Merelbeke, Belgium; 4 Ethiopian Food and Drug Authority (EFDA), Addis Ababa, Ethiopia

**Keywords:** Quality, Anti-malarials, Artemether, Lumefantrine, Jimma, Ethiopia

## Abstract

**Background:**

Malaria caused by *Plasmodium vivax and Plasmodium falciparum* is among the major public health problems in most endemic areas of the world. Artemisinin-based combination therapy (ACT) has been recommended as a first-line treatment for uncomplicated *Plasmodium falciparum* malaria almost in all endemic regions. Since ineffectively regulated medicines in resource limited settings could favour infiltration of poor quality anti-malarial medicines into pharmaceutical supply chain and jeopardize a positive treatment outcome, regular monitoring of the quality of anti-malarial medicines is critical. Thus, the aim of this study was to assess the quality of fixed dose combination (FDC) artemether (ART)/lumefantrine (LUM) tablets available in Jimma zone, Ethiopia.

**Methods:**

This study was conducted in Jimma zone, Ethiopia. A total of 74 samples of FDC ART/LUM (20 mg ART/120 mg LUM) tablets were collected from 27 public facilities. All samples were subjected to visual inspection and the relevant information was recorded. The samples were transported to Jimma University Laboratory of Drug Quality (JuLaDQ) and stored at ambient temperature (20 °C to 25 °C) until analysis. The Pharmacopoeial conform/non-conform methods and the risk-based Derringer’s desirability function approach were employed to assess the pharmaceutical quality of the investigated products.

**Results:**

The visual inspection results revealed that there were no signs of falsified in the investigated products. Identification test results of samples indicated that all samples contained the stated active pharmaceutical ingredients (APIs). The results of uniformity of mass indicated that all samples complied with International Pharmacopoeial specification limits. The assay results, expressed as percent label claim (%lc) of ART (89.8 to 108.8%, mean ± SD = 99.1 ± 3.9%) and LUM (90.0 to 111.9%, mean ± SD = 98.2 ± 3.8%) revealed that, all samples complied with International Pharmacopoeia acceptance specification limits (i.e. 90–110%lc), except one generic product (IPCA Laboratories Ltd., India) which contains excessive LUM (111.9 ± 1.7%lc). The risk priority number (RPN) results revealed that assay (RPN = 392) is relatively the most critical quality attribute followed by identity (RPN = 280) and mass uniformity (40). Quality evaluation based on psycho-physical Harrington’s scale revealed that more than 96% of samples were within the acceptable ranges (D ≥ 0.7–1.0).

**Conclusions:**

Both Pharmacopoeial and risk-based desirability function approaches to quality evaluation applied to the investigated products revealed that above 96% FDC ART/LUM tablets circulating in public settings of Jimma zone are of good quality.

**Electronic supplementary material:**

The online version of this article (10.1186/s12936-019-2872-1) contains supplementary material, which is available to authorized users.

## Background

Malaria is the major public health problem in Africa which accounts for 90% of all malaria cases and 92% of deaths ascribed to malaria in the world [1]. Interventions such as use of anti-malarial medicines, insecticide-treated bed nets and indoor residual sprays have been employed in malaria endemic areas [2–7] and reduced malaria prevalence by 50% and clinical incidence by 40% between 2000 and 2015 [3]. However, prevalence of poor quality anti-malarial medicines [8–10] linked to reduced efficacy, treatment failure or death [11–13] and insecticide resistance of malaria transmitting mosquitoes [14, 15] are obstacles potentially threatening the global target to eliminate incidence of malaria infection [16].

In Ethiopia, malaria infection caused by *Plasmodium vivax* and *Plasmodium falciparum* is a common problem that affects a large number of people living in malaria-endemic areas [17, 18]. Thus, to reduce mortality and morbidity due to malaria, fixed dose combination (FDC) artemether (ART)/lumefantrine (LUM) and chloroquine have been used as first-line treatment for malaria caused by *Plasmodium falciparum* and *Plasmodium vivax*, respectively [19]. Since poor quality [i.e. falsified (medical products that deliberately/fraudulently misrepresent their identity, composition or source) or substandard (authorized medical products that fail to meet either their quality standards or their specifications, or both) of anti-malaria medicines is one of the risk factors that could affect the intended clinical outcomes, ensuring the quality of medicines is crucial in providing quality health care services. In Ethiopia, though there are previous studies indicating the occurrence of poor quality of anthelminthic, anti-protozoal and non artemisinin-based combination therapy (ACT) anti-malarial medicines [20–24], no evidenced information is found on the quality of FDC ART/LUM products. Thus, evaluating the quality of FDC ART/LUM products circulating in the health facilities is critical to reduce risk of having poor quality medicines. Therefore, the aim of this study was to assess the pharmaceutical quality of FDC ART/LUM tablets available in Jimma zone, Oromia Regional state, Ethiopia.

## Methods

### Study area

**Fig. 1:**
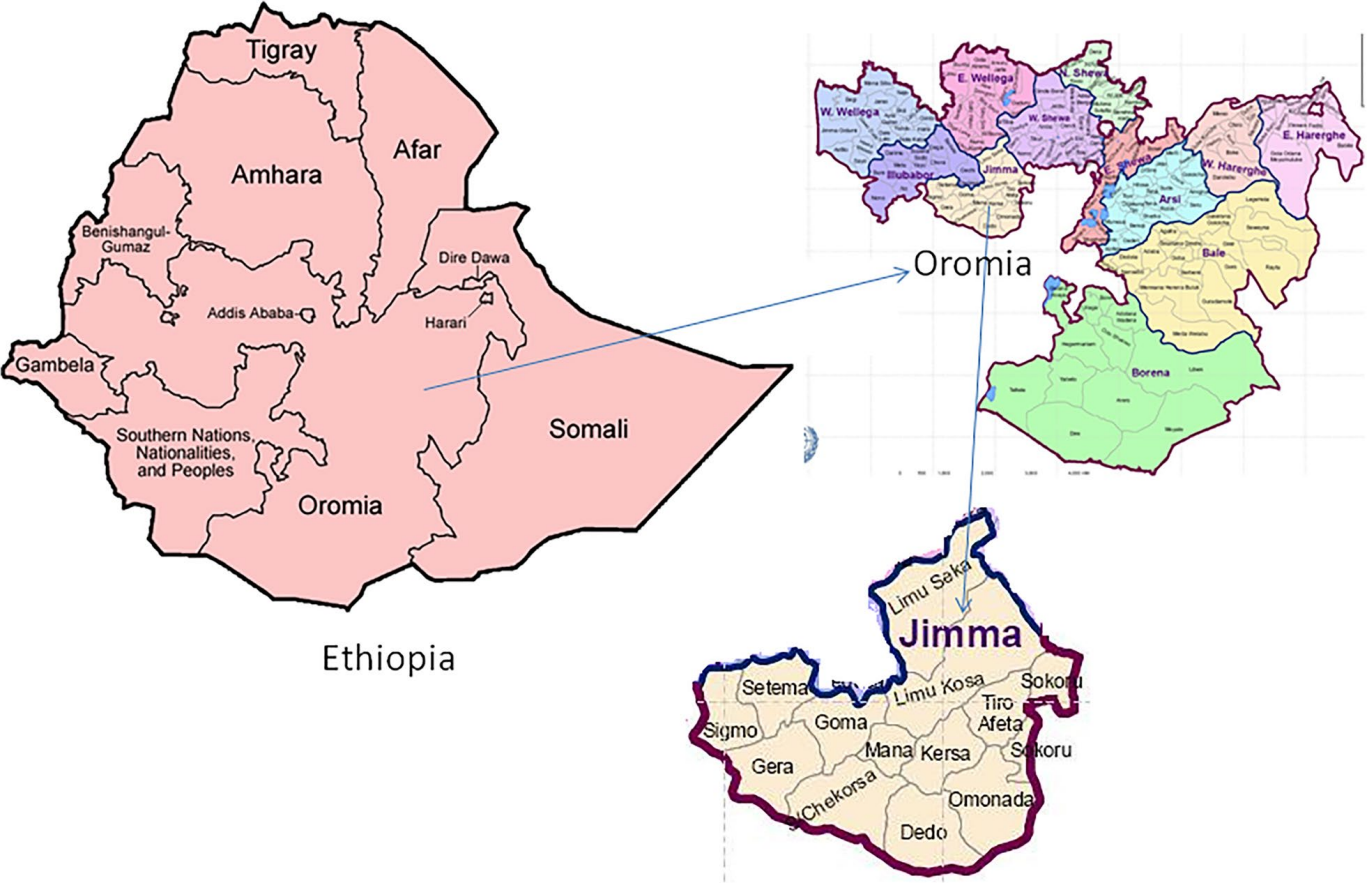
Map of Jimma zone, Oromia Regional State

The study was conducted in all districts of Jimma zone, Oromia Regional State. The areas were selected due to the fact that they are endemic for malaria. In Ethiopia, patients can get FDC ART/LUM products free of charge from public health centres and hospitals. Thus, these products are less likely to be dispensed in private drug retail outlets. Therefore, all government owned public facilities (health centre, hospitals and wholesales) operating in the study area were included in this study. Jimma is the commercial hub for the south west Ethiopia and thus relatively huge pharmaceutical transaction occurs in the region. Currently, there are 7 wholesales (all of them in Jimma city), 71 drug shops (31 in Jimma city and 40 in districts of Jimma zone), 101 public drug shops in health centres (3 in Jimma city and 98 in districts of Jimma zone), 18 private pharmacies (all of them in Jimma city) and 4 hospital pharmacies in Jimma zone. The map of Jimma zone, Oromia Regional State is presented in Fig. 1.

### Chemicals/reagents/solvents

Artemether (99% w/w) and lumefantrine (99% w/w) working standard active pharmaceutical ingredients (APIs) were supplied by Dafra Pharma International (B-2300 Turnhout, Belgium) through Drug Quality and Registration (DruQuaR) laboratory, University of Ghent, Belgium. Ultrapure water (18.2 MΩ cm at 25 °C) was prepared in Jimma University Laboratory of Drug Quality (JuLaDQ) using ultrapure water purification system (Thermofischer Scientific, USA). Methanol (HPLC grade, Fisher Scientific), acetonitrile (HPLC grade, Fisher Scientific), ethyl acetate and acetone (Fisher Scientific), glacial acetic acid and sulfuric acid (Reagent Chemical Services), sodium hexanesulfonate (Fluka Analytical, Germany) and sodium dihydrogen phosphate (Reagent Chemical Services) were used as received.

### Sample collection

The quality survey was conducted by considering the guidelines for field surveys of the quality of medicines proposed by Newton et al. [25]. A total of 74 FDC ART/LUM (20 mg ART/120 mg LUM) tablets samples were collected between May and June, 2013 from all public health facilities (where available) [wholesales (n = 2), hospital pharmacy (n = 1) and health centre drug stores (n = 24)] operation in Jimma zone. Sample with different products names (i.e. Coartem^®^ = 35 and generic = 39) and countries of origin [Ethiopia (n = 1), China (n = 1), India (n = 38) and USA (n = 34)] were collected anonymously by mystery shoppers from local area who were trained before to present a confirmed *P. falciparum* malaria patient of 25 years of age with a prescription stating 4 tablets twice per day for 3 consecutive days (full adult dose of 24 tablets). The mystery shoppers were blinded about the purpose of the study and only instructed to collect samples. The relevant information of all collected samples (level of drug outlet, place of collection, name of APIs, country of origin, manufacturing company, expiry date, manufacturing date, batch/lot number and strength of APIs) was recorded on standard form as soon as leaving the drug outlet and entered into database. The samples were transported to JuLaDQ and stored at ambient temperature (20 °C to 25 °C) until analysis.

### Physico-chemical quality assessment

All samples were visually inspected for physical characteristics of tablets (i.e. shapes, colour, breaks, cracks and splits), packaging and labelling information (i.e. name of the active pharmaceutical ingredient, the country of origin, manufacturing company, expiry date, manufacturing date, batch/lot number, number of units per strip/package and labelled dose (strength) of the active ingredient) using the checklist set by WHO [26]. The modified checklist is presented in Additional file 1.

### Identification tests

The presence of ART and LUM APIs in FDC ART/LUM tablets was analysed according to the method given in the International Pharmacopoeia [27]. In addition, thin layer chromatography, chromatographic peak retention time and DAD-UV absorption spectra were used for the purpose of identification through comparison with retention time and DAD-UV absorption spectra of the peak obtained on a working standard solutions of ART and LUM APIs.

### Uniformity of mass

The mass uniformity test of tablet samples was conducted according to the method given in the International Pharmacopoeia [28]. Randomly selected tablets (n = 20) of each sample were individually weighed with a calibrated balance (Mettler Toledo, AL204-1C, Switzerland) with an accuracy of 0.006%. The uniformity of mass of the tablet samples was evaluated against Pharmacopoeial specification (i.e. the deviation of individual masses of minimum of 18 and maximum of 2 tablets should not exceed by ± 7.5% and ± 15% from average mass, respectively).

### Assay of active substance in the tablet samples

The amount of ART and LUM APIs in samples of FDC ART/LUM tablets was determined based on the method given in an individual monograph of International Pharmacopoeia [29].

#### System suitability

System suitability was evaluated according to the European Pharmacopoeia method [30]. The symmetry factor (A_s_) of principal peaks was calculated using the following formula:

$$As = {W_x}/2d$$

where W_x_ = peak width at 5% of reference standard peak height measured from the base line, d = base line distance between the perpendicular dropped from the peak maximum and the leading edge of the peak at 5% of peak height measured in the same unit as W_x_. The specification was an A_s_ value of maximally 1.5. In addition, percent relative standard deviation (%RSD) of replicate injections (n = 6) of reference standards were calculated and compared against the European Pharmacopoeia specification limit, i.e. %RSD of sextuplicate injections should be maximally 2.

#### Mobile phase preparation

The mobile phases 70/30% v/v (A) and 30/70% v/v (B) ion pair reagent/acetonitrile (HPLC grade, Fisher Scientific) were prepared for gradient elution. Ion-pair reagent was prepared by dissolving 5.65 g of sodium hexanesulfonate (Fluka Chemicals Ltd.) and 2.75 g of sodium dihydrogen phosphate (Fishers Scientific) in 900.0 ml of ultra-pure water (18.2 MΩ cm) and adjusted to pH 2.3 using phosphoric acid (ReAgent Chemicals, UK), diluted to volume (1000.0 ml) and filtered through a 0.45 µm filter (Macherey–Nagel, Germany).

#### Working standard solution

Working standards of ART (20 mg) and LUM (120 mg) were individually added into 100.0 ml volumetric flask, dissolved in 85 ml of solvent (i.e. ion pair/ultra-pure water/1-propanol/acetonitrile: 20/6/20/54% v/v), sonicated, allowed to cool to room temperature and diluted to volume.

#### Sample solution

Twenty tablets of each sample were randomly selected and powdered. A portion of powder equivalent to 20 mg ART and 120 mg LUM was individually added into 100.0 ml volumetric flask, dissolved in 85.0 ml of solvent (i.e. ion-pair/ultra-pure water/1-propanol/acetonitrile: 20/6/20/54% v/v), sonicated (20 min), allowed to cool to room temperature and diluted to volume.

#### HPLC method

The analysis of samples was conducted using Agilent 1260 Infinity Series HPLC system (Agilent Technologies, Santa Clara, California, USA) equipped with a C18 column (Symmetry^®^) (150 mm × 3.9 mm, 5 μ particle size) coupled to a diode-array detector (DAD). The sample temperature in auto-injector was 25 °C. The column temperature, flow rate, injection volume, run time were 25 °C, 1.3 ml/min, 20 µl and 55 min, respectively.

### Risk analysis

Failure mode effects analysis (FMEA) was used to evaluate criticality of product quality attributes. Criticality was evaluated based on risk priority number (RPN) which considered occurrence, severity and detection of the failure and calculated using the formula:

$$RPN = O \times S \times D$$

where O, S and D denote the occurrence of a failure mode, the severity of a failure effect, and the probability of not detecting the failure, respectively [31].

The scales used for scoring severity, occurrence and detectability are presented in Tables 1, 2, 3.

**Table 1:** Traditional FMEA scale for occurrence. Source: Ford Motor Company. Potential Failure Mode and Effects Analysis (FMEA) Reference Manual; Ford Motor Company: Dearborn, MI, USA, 1988. https://www.worldcat.org/title/potential-failure-mode-and-effects-analysis-fmea-reference-manual/oclc/43210773

Probability of failure	Possibility of failure rates	Rank
Extremely high: failure almost inevitable	≥ 1 in 2	10
Very high	1 in 3	9
Repeated failures	1 in 8	8
High	1 in 20	7
Moderately high	1 in 80	6
Moderate	1 in 400	5
Relatively low	1 in 2000	4
Low	1 in 15,000	3
Remote	1 in 150,000	2
Nearly impossible	≤ 1 in 1,500,00	1

**Table 2:** Traditional FMEA scale for severity. Source: Ford Motor Company. Potential Failure Mode and Effects Analysis (FMEA) Reference Manual; Ford Motor Company: Dearborn, MI, USA, 1988. https://www.worldcat.org/title/potential-failure-mode-and-effects-analysis-fmea-reference-manual/oclc/43210773

Effect	Criteria: severity of effect	Rank
Hazardous	Failure is hazardous, and occurs without warning. It suspends operation of the system	10
Serious	Failure involves hazardous outcomes and/or noncompliance with government regulations or standards	9
Extreme	Product is inoperable with loss of primary function. The system is inoperable	8
Major	Product performance is severely affected but functions. The system may not operate	7
Significant	Product performance is degraded. Comfort or convince functions may not operate	6
Moderate	Moderate effect on product performance. The product requires repair	5
Low	Small effect on product performance. The product does not require repair	4
Minor	Minor effect on product or system performance	3
Very minor	Very minor effect on product or system performance	2
None	No effect	1

**Table 3:** Traditional FMEA scale for detection. Source: Ford Motor Company. Potential Failure Mode and Effects Analysis (FMEA) Reference Manual; Ford Motor Company: Dearborn, MI, USA, 1988. https://www.worldcat.org/title/potential-failure-mode-and-effects-analysis-fmea-reference-manual/oclc/43210773

Detection	Criteria: likelihood of detection by design control	Rank
Absolute uncertainty	Design control does not detect a potential cause of failure or subsequent failure mode; or there is no design control	10
Very remote	Very remote chance the design control will detect a potential cause of failure or subsequent failure mode	9
Remote	Remote chance the design control will detect a potential cause of failure or subsequent failure mode	8
Very low	Very low chance the design control will detect a potential cause of failure or subsequent failure mode	7
Low	Low chance the design control will detect a potential cause of failure or subsequent failure mode	6
Moderate	Moderate chance the design control will detect a potential cause of failure or subsequent failure mode	5
Moderately high	Moderately high chance the design control will detect a potential cause of failure or subsequent failure mode	4
High	High chance the design control will detect a potential cause of failure or subsequent failure mode	3
Very high	Very high chance the design control will detect a potential cause of failure or subsequent failure mode	2
Almost certain	Design control will almost certainly detect a potential cause of failure or subsequent failure mode	1

Pharmaceutical experts working at JuLaDQ (n = 3) and Ethiopian Food and Drug Authority (EFDA) (n = 2) were assigned to score for severity, occurrence and detectability. The potential effect of each failure mode was considered to assign a severity rating. Thus, the score of 10, 8 and 5 was assigned for identity, assay and mass uniformity, respectively. Literature data on prevalence of substandard and/or falsified anti-malarial medicines in Africa [8–10, 32] and the findings from this study were used to assign scores for occurrence. Therefore, the occurrence score of 7, 7 and 4 were assigned for identity, assay and uniformity of mass, respectively. Detecting defects of the mass uniformity and identity is relatively easy and does not require high technology facility, advanced knowledge and skills. Therefore, detectability score of 4 and 2 was assigned for identity and mass uniformity, respectively. On the other hand, since assay requires fully equipped laboratory system and advanced training of personnel, detectability score of 7 was assigned for assay. The assigned scores for severity, occurrence and detectability are presented in Additional file 2.

### Desirability function

Derringer’s desirability function [23, 33] was applied to assess quality of the evaluated product. The overall desirability function (D) value which is the geometric mean of the individual desirabilities (d_i_), indicates the product quality. The highest global desirability value represents the product with the highest quality. The D value was calculated using the following formula:

$$\sqrt[n]{{\mathop \prod \limits_{i = 1}^{n} {d_i}^{{p}_{i}} }}$$

where p_i_ is the weight or relative importance assigned to the response, n is the product quality attributes evaluated, d_i_ is an individual desirability. In this study, three product quality attributes (i.e. identity, assay and mass uniformity) were considered and thus n equals to 3 is taken in global evaluation of the investigated FDC ART/LUM tablet samples. According to RPN value, which is the numeric assessment of risk assigned to each quality parameter, p value of 3, 2 and 1 was assigned for assay, identity and mass uniformity, respectively.

Modified psychophysical five interval scale of Harrington’s desirability function [34] was constructed and used as a tool for quality judgment; i.e. qualitative assessments “bad”, “low”, “acceptable”, “good” and “excellent” which correspond to numeric intervals of 0.00–0.37, 0.37–0.70, 0.70–0.80, 0.80–0.90, and 0.90–1.00, respectively. The numeric desirability values of the quality attributes were considered as physical parameters; while the subjective judgments of the experts (i.e. excellent, good, acceptable, low or bad) were considered as psychological. For assay a two-sided desirability function was used; and a one-sided desirability function was used for identity and mass uniformity. Since absence of API is assumed to be clinically completely undesirable, d  =  0 was assigned. While d  =  1 was assigned for 100%lc which is assumed to be optimum desirability. Since assay of both ART and LUM APIs in FDC ART/LMU tablet should be from 90 to 110%lc [29] and psychophysical Harrington’s scale specifies desirability range from 0.70 to 1.00 to be good, d  =  0.7 was assigned for assay values of 90 and 110%lc and d  =  0.3 was assigned for 70% and 130%lc. While d  =  0.01 was assigned for 50% and 150%lc. The individual desirability function for assay was then defined as different linear sections of different slopes in the range of 100%lc to 90%lc (slope  =  0.03), 90%lc to 70%lc (slope  =  0.02) and from 70%lc to 50%lc (slope  =  0.01).

For mass uniformity, the relative standard deviation (RSD) was considered as response. According to European Pharmacopoeia [28], RSD should not be more than 2% and thus d  =  1, d  =  0.7, d  =  0.3, d  = 0.01 and d = 0 were assigned for RSD  of  0, 2, 6, 15 and 25%, respectively. For identity, d =  1.0 was assigned for samples complied with Pharmacopoeia specification. While d = 0 was assigned for those which do not comply.

## Results

A total of 74 samples (i.e. Coartem^®^ = 35, Artefan^®^ = 6, Artemine^®^ = 1, and artemether–lumefantrine = 32) were collected between May to June, 2013 from wholesales (n = 2), hospitals (n = 4) and health centres (n = 68).

The results of visual inspection of samples for physical characteristics of tablets, packaging and labelling information as set by WHO revealed that there were no signs of falsified in the investigated products. Detail information of samples on physical characteristics of tablets, packaging and labelling information is presented in Additional file 3.

Identification test results of samples revealed that all samples had the intended APIs as demonstrated by the positive identification tests. Consistent results were obtained with the Pharmacopoieal methods, thin-layer chromatography, HPLC retention and DAD information.

The results of uniformity of mass indicated that all samples complied with International Pharmacopoeia specification limits (i.e. the deviation of individual masses of minimum of 18 and maximum of 2 tablets should not exceed by ± 7.5% and ± 15% from average mass, respectively). The mass %deviation distributed among brand products are presented in Table 4.

**Table 4:** Results of mass %deviation distributed among brand products of FDC ART/LUM tablets

#	Product name (n)	%Deviation		
		Minimum	Maximum	Mean
1	Coartem^®^ (35)	0.75	7.11	1.61
2	Artemether–lumefantrine (32)	0.80	2.64	1.78
3	Artefan^®^ (6)	0.99	4.5	2.55
4	Artemine^®^ (1)	1.34	1.34	1.34

The results of amount of ART and LUM in the FDC ART/LUM tablet samples revealed that except one generic product (IPCA Laboratories Ltd., India) which failed to comply (111.9%lc) for LUM API, all samples complied with the Pharmacopoieal acceptance specification limit [i.e. 90.0–110.0% (percent label claim (%lc)]. The amount of ART API in samples analysed ranges from 89.8 to 108.8% (mean: 99.1%, SD: 3.9%), while that of LUM ranges from 90.0 to 111.9% (mean: 98.2%, SD: 3.8%). The amount of ART and LUM APIs in FDC ART/LUM tablet samples collected from health centre (ART = 89.78–105.92%, LUM = 91.19–111.87%), hospitals (ART = 97.70–101.94%, LUM = 90.03–104.24%) and wholesales (ART = 100.52–108.78%, LUM = 93.68–107.98%) indicated relative differences in the amount of ART/LUM APIs among investigated products. The results of the amount (%lc) of ART and LUM APIs in FDC ART/LUM tablet samples are presented in Additional file 4. Amount (%lc) of ART and LUM APIs distributed among brand and generic product is given in Table 5.

**Table 5:** Amount (%lc) of ART and LUM APIs distributed among brand and generic products

#	Product name (n)	API	%lc				
			Minimum	Maximum	Mean	SD	Median
1	Coartem^®^ (35)	ART	90.9	103.6	98.9	3.0	99.6
		LUM	90.0	104.1	97.4	3.4	97.7
2	Artemether–lumefantrine (32)	ART	91.5	106.7	99.2	4.3	99.7
		LUM	93.6	111.9	98.8	3.8	98.7
3	Artefan^®^ (6)	ART	89.8	103.4	98.2	5.0	99.4
		LUM	91.2	104.2	98.7	4.7	99.6
4	Artemine^®^ (1)	ART	108.8	108.8	108.8	NA	108.8
		LUM	108.0	108.0	108.0	NA	108.0

*SD* standard deviation, *NA* not applicable

### Risk analysis

The results of the RPN are presented in Table 6. Assay (RPN  =  392) is the most critical quality attribute followed by identity (RPN  =  280) and mass uniformity (RPN = 40).

**Table 6:** Failure mode and effect analysis for FDC ART/LUM tablet quality attributes

Critical quality attributes	Failure mode	Failure effects	Severity	Occurrence	Detection	RPN
Identity	No (intended) active ingredient in the sample or mislabelling (incorrect, inadequate or incomplete identification)	Treatment failure, death due to untreated disease, toxicity	10	7	4	280
Assay	Under-dose, over-dose	Treatment failure, toxicity due to over-dose, drug resistance due to under-dose	8	7	7	392
Uniformity of mass	Non-uniform distribution of dose/content within the individual dosage units	Sub-optimal therapy for a patient taking the sub-standard dosage unit and drug resistance	5	4	2	40

*RPN* risk priority number

### Derringer’s desirability function

**Fig. 2:**
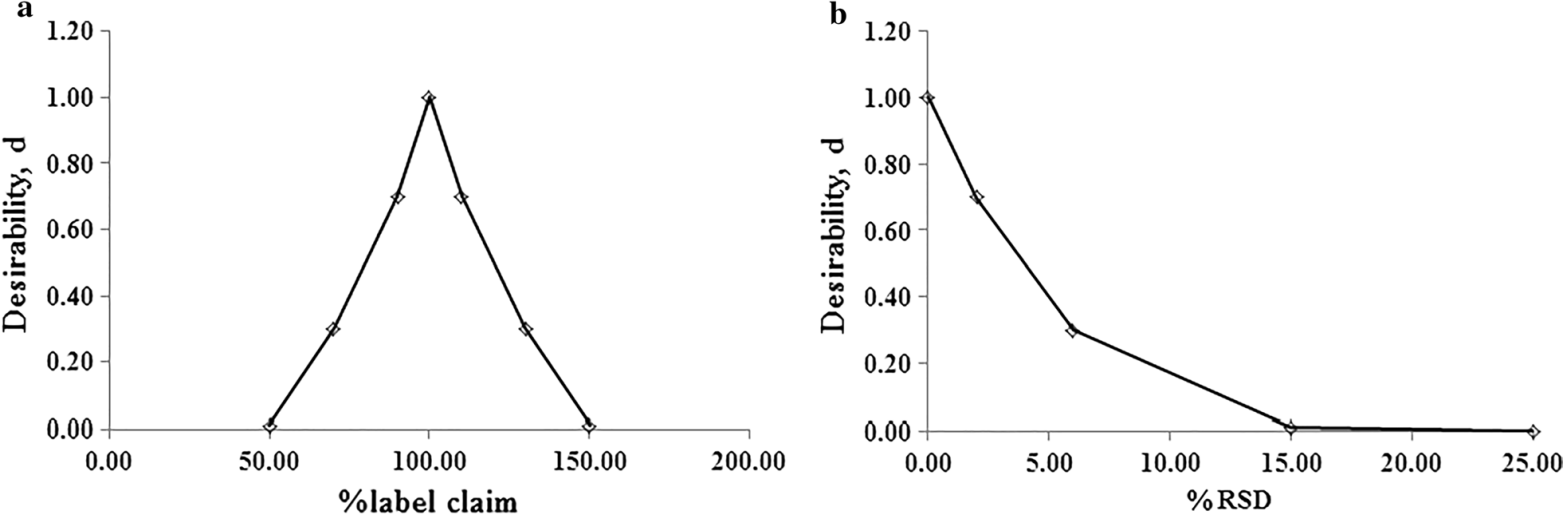
Linear desirability functions: **a** assay and **b** mass uniformity

The results of individual desirability values (d_i_) and the overall desirability (D) are presented in Additional file 5. The individual desirability values assigned to the different segments were fitted to the segmented linear model as presented in Fig. 2.

A global D-value of investigated products was calculated using d-functions of quality attributes (i.e. identity, assay and mass uniformity). Since FDC ART/LUM tablet contains FDC 20 mg ART and 120 mg LUM APIs, d-functions of identity and assay for ART and LUM were calculated individually. Based on Harrington’s scale of quality, 96% of the investigated products were within acceptable range (D = 0.7–1.0). The results of quality evaluation based on modified psycho-physical Harrington’s scale of quality are presented in Table 7.

**Table 7:** Modified psycho-physical Harrington’s scale of quality

#	Interval in global desirability	Quality, descriptive evaluation	Percent of products in each quality scale
ART in FDC ART/LUM tablets			
1	0.90–1.00	Excellent	47.3
2	0.80–0.90	Good	31.1
3	0.70–0.80	Acceptable	20.3
4	0.37–0.70	Low	1.4
5	0.00–0.37	Bad	–
LUM in FDC ART/LUM tablets			
1	0.90–1.00	Excellent	39.2
2	0.80–0.90	Good	36.5
3	0.70–0.80	Acceptable	20.3
4	0.37–0.70	Low	4.1
5	0.00–0.37	Bad	–

## Discussion

In this study, the pharmaceutical quality of FDC ART/LUM tablets was assessed. The results of visual inspection on physical characteristics of tablets, packaging and labelling information revealed that none of the samples demonstrated signs of falsified as defined by the WHO [35]. In addition, packaging of the investigated products complied with the WHO guideline on packaging for pharmaceutical products [36]. Appropriate packaging is important to protect the pharmaceutical products from environmental and transportation stress which are risk factors for product quality defects. Thus, the investigated products might not have a risk of stability problems associated with packaging [37].

The identity test results indicated that none of the samples contained incorrect APIs which revealed that due to the investigated products there was no risk of treatment failures or death [38–40]. The study results indicated that the investigated products complied with mass uniformity specification set in International Pharmacopoiea for uncoated and film-coated tablets formulated to contain 5% or more of the active ingredient (i.e. the deviation of individual masses of minimum of 18 and maximum of 2 tablets should not exceed by ± 7.5% and ± 15% from average mass, respectively) [28]. This suggests that variations observed in mass among dose units in each batch might not affect the content uniformity of the dose units of the investigated products as mass uniformity is an alternative test for content uniformity of uncoated and film coated tablets.

The results of amount of APIs indicated that majority (98.6%) of investigated products comply with Pharmacopoeial specification limit (i.e. %lc = 90–110%) [29]. Only one generic product contain excess LUM API (111.7%lc) and thus failed to comply with International Pharmacopoeia (Ph. Int.) specification. The results of amount of APIs suggest that the dose units of majority of the investigated products contain sufficient levels of both ART and LUM APIs and could not have a risk of treatment failure [12, 40–42], longer parasite clearance time and greater recrudescence [43, 44] and emergence of resistance [13]. Though effectiveness of medicines depends on various factors such as food intake, age, nutritional status, pharmacokinetic and IC50 of parasites [45, 46], sufficient amount of ART and LUM APIs observed in majority of the investigated products is very critical to reduce asexual parasite mass, eliminate the residual parasites and prevent recrudescence [47] and augment the recommended parasitological and clinical cure rate (≥ 90%) [48] The presence of excess LUM API (111.7%lc) in one generic product having the same batch number with the products which contain LUM API ranging from 96.1 to 102.0%lc could be attributed to excipient particle size, blending techniques or processing parameters that could influence product quality [49]. This points to poor adherence to Good Manufacturing Practice (GMP).

According to the WHO, multisource products must satisfy the same standards of quality, safety and efficacy applied to the corresponding innovator product [50]. In this study, majority of investigated products qualify Pharmacopoeial specification limits set for amount of API. Thus, interchangeable use of these products may not have a risk arising from amount of APIs.

The use of poor quality anti-malarial medicines could have a risk of reduced efficacy, an increased development of drug resistance, prolonged and more severe illness or unexpected adverse effects [11, 12, 32, 42, 43, 51]. Therefore, evaluating criticality of product quality attributes for efficacy using risk analysis method is crucial for risk mitigation strategies. To this end, failure mode effects analysis (FMEA) method [52] was used and the risk associated with each failure mode (identity, assay or mass uniformity) was evaluated based on the values of RPN. As a result, assay (RPN = 392) is found to be the most critical quality attribute followed by identity (RPN = 280) and mass uniformity (RPN = 40). This implies that quality risk with regard to assay is relatively higher.

Compared to conventional (Pharmacopoieal) method of quality verification, risk-based desirability function approach to quality evaluation provides more weight to the clinically more critical quality attributes. For products having various quality characteristics, the quality of the product is completely unacceptable if one of the characteristics lies outside the desired limits. In this study, the quality of investigated products was evaluated based on calculated D values compared against constructed numeric intervals ranging between 0 and 1. Thus, qualitative assessments of the investigated products (bad, low, acceptable, good and excellent) which correspond to respective numeric intervals were conducted based on modified psycho-physical Harrington’s scale of quality. Based on the results obtained from risk based desirability approach of quality evaluation and considering ART and LUM APIs, 96% and 98.7% of the quality of the investigated products lie within acceptable range (0.70–1.00), respectively. The results of risk-based desirability approach quality evaluation are in-line with the results (98%) observed in Pharmacopoeial (conform/non conform) quality verification approach. Since using of risk based desirability approach provides more weight to clinically more critical quality attributes, this approach helps to make reliable decision based on clinically more critical quality attributes. In addition, it could be used to determine deviations from the desired response levels. Moreover, risk based desirability approach of quality evaluation less heavily penalize marginal out-of specification medicines and assumed to save economic loss in resource scarce society.

## Conclusions

The results of this finding revealed that the quality of 96–98% of the investigated products lies within acceptance limit when evaluated using both Pharmacopoeial and risk-based desirability function approaches. This suggests that the medicines circulating in the public settings of Jimma zone are of good quality. However, this does not necessarily mean that there is effective regulatory system that helps to prevent infiltration of poor quality medicines into pharmaceutical supply chain. Therefore, complete nationwide survey which includes private settings should be done.

## Additional files

**Additional file 1.** Checklist. Physical characteristics, packaging and labelling information.

**Additional file 2.** Assigned scores for each factor of failure modes.

**Additional file 3.** Physical characteristics, packaging and labelling information.

**Additional file 4.** Results of the amount of ART/LUM in FDC tablets.

**Additional file 5.** Desirability function values.

## Data Availability

The datasets used and/or analysed during the current study are available from the corresponding author on reasonable request.

## Abbreviations

ART: artemether
LUM: lumefantrine
FDC: fixed dose combination
ACT: artemisinin-based combination therapy
WHO: World Health Organization
ICH: The International Conference on Harmonization
