# Supplementary material for: Quality of fixed dose artemether/lumefantrine products in Jimma Zone, Ethiopia
Source: Malar J. 2019 Jul 15;18:236. doi: 10.1186/s12936-019-2872-1 (PMC6628471; doi:10.1186/s12936-019-2872-1)
Supplement: Supplementary file 2 — Additional file 2. Assigned scores for each factor of failure modes. [file 12936_2019_2872_MOESM2_ESM.docx]

**Assigned scores for factors of the evaluated failure modes of FDC ART/LUM tablet**.

| **Experts** | **Identity** | | |
| --- | --- | --- | --- |
|  | **Severity** | **Occurrence** | **Detectability** |
| SS | 9 | 8 | 5 |
| SB | 10 | 8 | 5 |
| GZ | 10 | 3 | 5 |
| GG | 10 | 8 | 3 |
| BZ | 9 | 9 | 4 |
| **Experts** | **Assay** | | |
|  | **Severity** | **Occurrence** | **Detectability** |
| SS | 9 | 8 | 8 |
| SB | 8 | 8 | 8 |
| GZ | 7 | 7 | 7 |
| GG | 8 | 7 | 7 |
| BZ | 9 | 7 | 7 |
| **Experts** | **Mass Uniformity** | | |
|  | **Severity** | **Occurrence** | **Detectability** |
| SS | 5 | 2 | 4 |
| SB | 4 | 3 | 1 |
| GZ | 6 | 5 | 3 |
| GG | 6 | 5 | 2 |
| BZ | 6 | 6 | 2 |
